# Towards a Food-Based Intervention to Increase Protein Intakes in Older Adults: Challenges to and Facilitators of Egg Consumption

**DOI:** 10.3390/nu10101409

**Published:** 2018-10-02

**Authors:** Emmy van den Heuvel, Jane L. Murphy, Katherine M. Appleton

**Affiliations:** 1 Research Centre for Behaviour Change, Department of Psychology, Faculty of Science and Technology, Bournemouth University, Poole BH12 5BB, UK; mvandenheuvel@bournemouth.ac.uk; 2 Faculty of Health and Social Sciences, Bournemouth University, Bournemouth BH1 3LT, UK; jmurphy@bournemouth.ac.uk

**Keywords:** egg consumption, dietary protein consumption, older adults, questionnaires

## Abstract

Background: Dietary protein intake is important for health. Eggs, as a protein-rich food with characteristics that appeal to older adults, may provide opportunities for increasing protein intake. Interventions that focus on the challenges or facilitators that affect a large proportion of the population will be of increased impact on a population-wide scale. This work aimed to investigate the relative importance of a number of challenges to and facilitators of egg consumption in a UK population-wide sample of older adults. Methods: A cross-sectional postal questionnaire, measuring habitual egg intake, reasons for eating/not eating eggs and a range of demographic and lifestyle characteristics, was administered by post to 1082 older adults. Results: 230 questionnaires suitable for analysis were returned (110 females, ages 55–80+ years). Habitual egg intake ranged from 1–89 eggs/month, mean (standard deviation) = 18 (13) eggs/month. Reasons for eating/not eating eggs were reduced using Principal Components Analysis to 23 challenges and facilitators of egg consumption. Regression analyses revealed habitual egg intake to be associated with 10 challenges and facilitators (smallest *β* = 0.14, *p* = 0.04), and with protein consumption, age and Body Mass Index (smallest *β* = 0.14, *p* = 0.03). Discussion: Many possibilities for future intervention based on existing challenges or facilitators were found. Our results suggest that strategies to increase egg consumption in older adults should focus on: improving liking, tastiness and adding variety; promoting eggs as an everyday type of food; reducing stereotypes about who does and who does not consume eggs; and promoting eggs for people who have noticed the effects of ageing on their food intake. Strategies that highlight value-for-money may be counterproductive. Future work evaluating the value of these strategies for improving protein intake in this age group would be of value.

## 1. Introduction

The older population is rapidly increasing [1], thus, maintaining or improving health in later years is becoming increasingly important. Dietary protein intake has a considerable impact on health and physical functioning [2,3,4]. Protein intake has been associated with functional abilities [5], reduced risk of incident frailty [6,7], falls and fractures [8,9], decreased bone mineral density and bone mass [10,11,12,13], improved glucose control in type 2 diabetes [14], lower blood pressure and lower risk of coronary heart disease [15,16,17]. Moreover, increasing protein intake has been positively related to the prevention of [18], and recovery [19,20] from injury, and may positively influence muscle mass and function in the elderly [6,21,22,23,24,25,26].

Energy intake in adults decreases approximately 25% between the ages of 40 to 70 years old [27], and older adults are known to eat less protein than younger adults [28,29,30]. Protein-specific under-nutrition in older adults ranges from 1%–24% if based on the estimated average requirement, but it can be as high as 77% when higher intake recommendations are used [30,31,32,33].

Previous studies have shown that older adults face specific challenges in consuming and increasing dietary protein. Barriers to eating protein-rich foods include sensory characteristics, the physical abilities involved in food preparation and food shopping, and eating capabilities, e.g., in biting, chewing, or swallowing, [4,27,28,34,35]. It has also been shown that perceived convenience, value for money, and low perishability are important positive predictors of intakes of protein-rich foods in older adults [35], and that intakes of these foods in this population are strongly affected by familiarity with the foods, habits and past eating behaviour [34,36]. These latter findings highlight the potential relevance of a food-based approach for increasing protein intake.

Eggs are a nutrient dense, high quality source of protein [37,38]. Sensory analyses with older adults have demonstrated that eggs are popular for their soft texture, while meats are characterized to have more difficult textures [39]. Compared to other protein-rich foods, eggs are also easy to cook, have a long shelf-life and also have a low cost [40,41]. Moreover, eggs are a familiar food to many people [38]. Given these characteristics, eggs may help increase dietary protein intakes in older adults.

However, to date, little research has investigated egg consumption in older adults, or identified the specific challenges to consuming eggs for this population. To encourage consumption, details of the challenges are required. Interventions that either address challenges or maximise facilitators will have increased chances of success compared to interventions that are less developed [42,43]. Furthermore, interventions that focus on challenges or facilitators that have an impact on a large proportion of the population will be of increased impact on a population-wide scale [42]. Previous qualitative work by ourselves identified all the reasons given for eating and not eating eggs in a sample of 42 British older adults [44]. Sixty-nine different reasons for eating and not eating eggs were found, many related to both consumption and non-consumption in different individuals. The current study aimed to extend this work, to investigate the relative importance of each of these reasons for egg consumption in UK older adults on a population-wide basis.

## 2. Methods

The study was undertaken using a cross-sectional postal questionnaire administered to a national sample of adults from the UK over 55 years old. Agreement/disagreement with each of the previously identified reasons for egg consumption/non-consumption was assessed alongside habitual egg intake and various demographic and lifestyle characteristics. Relative importance of each reason was then attained using regression analyses. Ethical approval for this study was granted by the Research Ethics Committee of Bournemouth University prior to commencement (ID: 8171).

### 2.1. Questionnaire

The questionnaire contained a food frequency questionnaire (FFQ) to measure habitual egg intake, an FFQ to measure habitual intakes of other protein-containing foods, a series of statements on reasons for eating/not eating eggs and a range of demographic and lifestyle characteristics, in this order.

**Egg Intake:** The FFQ for eggs listed 18 different types of egg preparation: boiled eggs (hot), hard boiled eggs (cold), fried eggs, scrambled eggs, poached eggs, omelettes, scotch eggs, quiches/savoury flans, egg mayonnaise, egg sandwiches, egg salad, custards, meringues, sweet flan/crème caramel, duck/quail’s eggs, raw eggs, egg yolk separate from the white, and egg white separate from the yolk. Participants were asked to report frequency of consumption on a seven point scale of ‘more than once a day’, ‘more or less daily’, ‘3–5 days a week’, ‘1–2 days a week’, ‘1–3 days a month’, ‘less than monthly’, and ‘never’. FFQs have previously been demonstrated as valid measures of food intake in older individuals [45,46].

**Protein Intake:** The same FFQ measures were used to assess participants’ habitual intake of a list of 18 other protein-containing foods: white meat (e.g., chicken, turkey); red meat (e.g., beef, lamb, pork); processed meat (e.g., ham, bacon, sausages, corned beef); white fish (e.g., cod, haddock); oily fish (e.g., sardines, salmon); seafood (e.g., prawns, mussels, crab); vegetarian meat substitutes (e.g., Quorn); milk in coffee or tea; milk (excluding milk in tea/coffee); yoghurt, custards, blancmanges, etc.; hard cheeses (e.g., Cheddar, Stilton); soft cheeses (e.g., cream cheese, brie, cottage cheese); nuts and seeds; pulses (e.g., lentils, Dahl); beans or peas; bread (e.g., white or whole meal); and breakfast cereals or porridge. Intakes of other protein-containing foods were assessed to allow consideration of regular protein intakes in our analyses. The foods were selected based on other studies [35] and the sources of protein contributing most to protein intake in British older adults in the National Diet and Nutrition Survey (NDNS) data [29].

**Reasons for eating or not eating eggs:** The series of statements on reasons for eating/not eating eggs included at least one statement for each of the 69 reasons previously identified [44], resulting in a total of 76 statements. Seven reasons were queried using several statements because they referred to a combination of slightly different topics. For example, the reason ‘recommendations’ was represented with three statements relating to: recommendations from the media, recommendations from friends and family, and recommendations from health professionals. Nineteen statements were also repeated for ‘my favourite type of egg’ as well as given for ‘eggs’ in general. Eggs can be consumed using a number of different preparations and it was recognised that some of the reasons given for eating eggs in the qualitative work may be more relevant for some preparations than others. For example, the reason ‘effort to prepare’ may be low for eggs in general compared to other protein-rich foods, but may also be high for ‘omelettes’ compared to ‘raw eggs’. These questions were asked for ‘my favourite type of egg’ as opposed to all preparations to avoid participant burden, but participants were asked to indicate their favourite egg preparation. All statements were responded to on a five point Likert scale using the answer options: ‘strongly disagree’, ‘disagree’, ‘neither disagree nor agree’, ‘agree’, and ‘strongly agree’. For all statements, special attention was paid to the wording of the statements, through reference to the focus group transcripts [44], for the language used by the target population.

**Demographic and lifestyle characteristics:** Questions on demographic characteristics requested: gender; age group; height and weight (converted to Body Mass Index (BMI)); marital status; living status; (first half of) postcode; education level (total number of years); nationality; and most recent level of employment. Lifestyle factors requested were: allergies to eggs; conditions that may have changed eating behaviour (e.g., chemo/radiotherapy) in the last 6 months; difficulties with everyday activities (measured using an adapted version of the SARC-F questionnaire to give an indication of frailty or sarcopenia [47]); help received with food shopping or preparation; food deliveries or eating out or away from home; denture wearing; and food neophobia (a reluctance to try new foods) [48,49]. These demographic and lifestyle characteristics have previously been associated with food intake in older individuals, e.g., [27,31,32,33,34,35,48,49].

Prior to administration, a pilot study was performed to test the questionnaire for face validity and internal reliability. Fifteen individuals from the target age group were asked to complete the questionnaire in the presence of the researcher, while simultaneously providing observations and questions in a ‘think aloud’ style [50]. Question statements and formatting were refined during and following piloting to increase clarity.

### 2.2. Questionnaire Administration

The questionnaire was sent by post to a National sample of 1000 community-dwelling adults over 55 years old. The sample was representative of the number of males and females per 5 year age group living in each different area of the UK, as reported in the UK Census 2011 [51]. Names and addresses were obtained from the data sampling company Sample Answers, London, UK. An additional 82 questionnaires were also sent out to a National sample of people aged over 55 years old who had taken part in previous studies and indicated that they would be willing to be contacted again. All individuals were living in their own homes at the time of questionnaire administration. A total of 588 participants were pre-notified by telephone with a brief conversation or voicemail message announcing that a questionnaire would arrive at their address soon. Reminders were sent out to non-responders about 6 weeks and 6 months after posting the first questionnaire.

### 2.3. Analyses

First, questionnaires from respondents with allergies to eggs, or who had had conditions that had changed their eating behaviour in the last 6 months were excluded. Remaining questionnaires were then screened for missing and inappropriate values. If responses for habitual egg intake and the reasons for eating eggs were missing by more than 20% per respondent, this individual’s data were excluded from analyses. If 80% or more questions were completed, missing values were completed with the value for ‘neither disagree nor agree’, or an average score if two answers had been given. If responses for habitual egg intake were greater than three standard deviations above the mean, these participants were also excluded.

Gender, age and location for the sample were then examined using *χ*^2^ tests to assess whether the sample for analysis was representative of the British population over 55 years old according to the UK Census 2011 [51].

Multiple linear regression analyses were then used to predict habitual egg intakes using all reasons for eating/not eating eggs, taking into account demographic and lifestyle characteristics, including intake from other protein-containing foods.

Egg FFQ data were converted to one number representing number of eggs eaten per month, where the category ‘more than once a day’ was counted as 60 times per month, ‘more or less daily’ was counted as 30 times, ‘3–5 days a week’ as 16 times, ‘1–2 days a week’ as 6 times, ‘1–3 days a month’ as 2 times, ‘less than monthly’ as 0.5 times, and ‘never’ was counted as 0 times per month, for all dishes excepting egg mayonnaise, custards, meringues, and sweet flan/crème caramel. Responses to egg mayonnaise were discounted due to their small and inconsistent contribution to total egg intakes based on portion size and amount per portion (the question was useful and retained in the questionnaire for reasons of face validity) [29]. For the sweet dishes, each consumption was counted as 0.5 portions, because a standard portion tends to amount to less than one egg [29]. Protein FFQ data were converted similarly for all foods with the exception of milk in coffee or tea, which was included as 0.2 portions each time it was consumed [29].

Responses to all statements on reasons for eating/not eating eggs were coded from 1: ‘strongly disagree’ to 5: ‘strongly agree’. Considering the large number of statements (76 statements), a Principal Component Analysis (PCA) was then conducted to reduce the number of variables to be included in subsequent regression models [52]. The PCA was conducted on all suitable data collected from the initial administration and 6 week reminders of the questionnaire (*n* = 182), using an orthogonal rotation and varimax method [53]. To avoid over reduction, all components with an eigenvalue >1 were considered, and component loadings were used together with semantic reasoning to identify the contributors to each component. Component scores were then generated per individual by adding all relevant items. The PCA was conducted using all statements relating to ‘eggs’ in general. Only 102 participants completed the questions on ‘my favourite type of egg’. Due to this low response rate, these items were not considered further for analysis.

Significant demographic and lifestyle characteristics in this sample were also identified in advance of the main analyses using multiple linear regression, where habitual egg intake was predicted by all demographic and lifestyle characteristics, including intake from other protein-containing foods. This analysis was done in advance of the main analyses to maintain good power in the main analyses [52].

Finally, multiple linear regression analyses were undertaken to predict habitual egg intakes using all calculated PCA components, taking into account significant demographic and lifestyle characteristics. Data analyses were performed using IBM SPSS Statistics software version 22.0 (IBM, Armonk, NY, USA).

## 3. Results

### 3.1. Respondents

A total of 259 individuals returned the questionnaire (24% response rate). Of these, 29 questionnaires were excluded based on the presence of allergies, medical conditions and/or missing values. Demographic information for the remaining 230 respondents is given in Table 1.

The sample was representative of the UK Census 2011 in terms of gender (*χ*^2^(1) = 2.97, *p* > 0.05), but 55–59 year old and 80+ year groups were under represented, while the 65–69 year old, 70–74 year old, and 75–79 year old groups were over represented (*χ*^2^(6) = 37.36, *p* < 0.05), and for location, Scotland was under represented, and the South West was over-represented (*χ*^2^(12) = 60.15, *p* < 0.05).

### 3.2. Egg Intake

Habitual egg intake for the sample ranged from 1–89 eggs/month with a mean (SD) of 18 (13) eggs/month.

#### 3.2.1. Reasons for Egg Consumption—Principal Components Analysis

The PCA resulted in 23 factors explaining 69.9% of the variance. These 23 components were titled: liking/flavour/variety; value for money; food chain; everyday food; effort; previous experience; past; occasion; stereotypes; sensory; expectations; willingness to eat more eggs; external reports; eating less with aging; medical factors; time; social environment; non-habitual intake; moreish; suitability; familiarity; size; and food safety. Contributing reasons and definitions are given in Table 2. Minimum, maximum, mean and SD for all component scores are given in Table 3. Component scores in the final sample showed no multicollinearity (largest *r* = 0.60, *p* < 0.01).

#### 3.2.2. Demographic and Lifestyle Characteristics—Initial Analysis

Living status and marital status were highly correlated (*r* = 0.70, *p* < 0.01), so only living status was included in regression models. No other evidence of multicollinearity was found (largest *r* = 0.56, *p* < 0.01). The multiple linear regression model including all demographic characteristics and lifestyle factors significantly predicted egg intake (*r* = 0.41, *r*^2^ = 0.17, adjusted *r*^2^ = 0.10, F (15,217) = 2.69, *p* < 0.01). Egg intake was significantly associated with a higher intake of other protein-containing foods (*β* = 0.31, *p* < 0.01), a younger age (*β* = −0.16, *p* = 0.04), and a higher BMI (*β* = 0.16, *p* = 0.02). All standardised co-efficients (*β* values) and *p* values can be found in Table 4.

#### 3.2.3. Egg Intake, Reasons for Egg Intake and Significant Demographic and Lifestyle Characteristics

The final regression model significantly predicted egg consumption (*r* = 0.60, *r*^2^ = 0.36, adjusted *r*^2^ = 0.28, *F* (26,218) = 4.20, *p* < 0.01). Greater egg intakes were significantly associated with: greater liking and/or greater agreement that eggs are tasty and add variety to the diet (*β* = 0.22, *p* = 0.02); less agreement that eggs are good value for money (*β* = −0.25, *p* < 0.01); higher agreement that eggs are an everyday type of food (*β* = 0.23, *p* = 0.01); less agreement that a certain type of person eats eggs (*β* = −0.20, *p* = 0.01); greater agreement that eggs should be eaten in certain circumstances (*β* = 0.17, *p* = 0.02); lower willingness to increase egg intake (or greater agreement that sufficient quantities of eggs are already consumed) (*β* = −0.16, *p* = 0.01); greater agreement with eating less with aging (*β* = 0.25, *p* < 0.01); lower agreement that eggs may increase cholesterol or risk of heart disease (*β* = −0.15, *p* = 0.03); greater agreement with eating eggs as a treat and trying new recipes with eggs (*β* = 0.14, *p* = 0.04); greater difficulty stopping eating eggs once started (*β* = 0.16, *p* = 0.02); greater consumption of other protein-containing foods (*β* = 0.24, *p* < 0.01); a younger age (*β* = -0.16, *p* = 0.02); and a higher BMI (*β* = 0.14, *p* = 0.03). All standardised coefficients (*β* values) and p values can be found in Table 5.

## 4. Discussion

This work was conducted to elicit the most important challenges or facilitators associated with habitual egg intake in the older population, with a view to suggesting interventions to increase egg intakes in this group. Many challenges/facilitators were found, alongside associations with three demographic/lifestyle variables.

Strongest associations were found between a higher habitual egg intake and greater liking and/or greater agreement that eggs are tasty and add variety to the diet, higher agreement that eggs are an everyday type of food, less agreement that a certain type of person eats eggs and greater agreement with eating less with aging. Liking and flavour are known important determinants of eating behaviour in the elderly for a range of foods [34,54,55,56]. Variety is also well known to increase food intake [57,58,59,60,61]. Associations between egg intake and consideration of eggs as an everyday type of food are also unsurprising. Studies in younger adults have reported that eggs are considered a staple food [40,62] and in the UK in the 1960s, eggs were advertised using the slogan ‘Go to work on an egg’ [63], possibly aiding this perception in the current older population. Less agreement that a certain type of person eats eggs may also be linked to consideration of eggs as an everyday food. Alternatively, however, the stereotypes associated with egg consumption can be varied, ranging from consideration of eggs as a working class and masculine food, to a light and more feminine food [44,64]. Consumption of eggs was higher in those with less firm stereotypes. Older people have also previously been found to worry about consumption stereotypes [65], so less agreement with stereotypes may be preferable. Associations between egg consumption and eating less with ageing are also unsurprising. These findings suggest that older adults with more age-related difficulties with food preparation and shopping, or age-related deteriorations in sensory abilities and/or appetite tend to eat more eggs, and support suggestions that compared to other protein-rich foods, eggs may be particularly suitable for older adults with sensory or physical impairments [4,27,28,34,35].

Interventions that focus on existing challenges or facilitators will likely have increased impact [42,43]. These results then suggest that strategies to increase egg consumption should focus on: improving liking, tastiness and adding variety; promoting eggs as an everyday type of food; reducing stereotypes about who does and who does not consume eggs; and promoting eggs for people who have noticed the effects of ageing on their food intake. Studies have shown that improving flavour by adding spices, flavour enhancers or sauces can increase intakes of protein-rich foods in older adults [66,67,68,69]. The use of several different added flavours also provides the possibility of increasing the hedonic variety of the diet, and increasing variety has been found to increase intake in older adults [58]. The promotion of foods based on their sensory characteristics, such as taste and texture, is also a well-used strategy known to result in changes in food intakes [70,71,72]. Associations with agreement that eggs are an everyday type of food would suggest a benefit from promoting eggs in this manner. This type of promotion may benefit from focus on the flexibility, easy to use, easy storage and long shelf life of eggs [40,41]. Studies also show that changing perceptions of those who eat and do not eat certain foods can change attitudes and behaviours towards those foods [73,74]. Although stereotypes can be resistant to change in the natural environment [75], interventions using social norms and strategies like social modelling have been successful at changing eating behaviours in younger individuals [73,74]. Some sensitivity, however, may be required when promoting foods specifically for ageing individuals or individuals with disabilities. Many older individuals may not think of themselves as old, or may not like to think of themselves as in need of special consideration [76,77]. For these individuals, the promotion of eggs as a food suitable for ageing individuals may even be counterproductive. Given these considerations and the above concern for broadening or reducing stereotypes, greater success may be achieved by promoting eggs for all, based on taste, variety and everyday use, as above.

The association between habitual egg intake and less agreement that eggs are good value for money is surprising. Eggs have previously been suggested as good value for money based on protein quality and cost [37,38,40,41], and previous reports suggest that value for money can be an important determinant of food choice in older individuals [35,55,61]. It is unlikely that individuals who consume a lot of eggs are not aware of their value or cost, but it is possible that these individuals do not want to admit value- or cost-based judgements or do not want to be perceived as consuming eggs for value- or cost-based reasons. These findings would suggest that strategies that promote value-for-money may be counterproductive. Interestingly furthermore, when an exploratory regression analysis was undertaken involving only the component ‘value for money’, the model did not significantly predict egg intake (*r* = 0.074, *r*^2^ = 0.005, adjusted *r*^2^ = 0.001, *F* (1228) = 1.24, *p* = 0.27), suggesting that value for money only significantly explains variance in egg intake when the variance explained by the other components is also considered.

Smaller associations were also found between habitual egg intake and greater agreement that eggs should be eaten in certain circumstances or with certain other foods, lower willingness to increase egg intake (or greater agreement that sufficient quantities of eggs are already consumed), lower agreement that eggs are associated with cholesterol and cardiovascular disease risk, greater agreement that eggs can be consumed in a non-habitual manner, e.g., as treats, and greater difficulty stopping eating eggs once started. Findings related to expectations that eggs should be consumed in certain circumstances or with certain other foods are also possibly linked to perceptions of eggs as everyday foods (eggs may not be suitable for dinner parties), or the usual consumption of eggs in a familiar or habitual manner. Food intake can be strongly affected by familiarity and habit in older individuals [34,36,78]. These associations may again suggest the promotion of eggs as an everyday, familiar food. Associations, however, were also found between high egg intake and greater agreement with consuming eggs in a non-habitual manner, e.g., as treats. These findings may suggest that there are two different types of high egg consumer—those who consume a lot of eggs on a habitual basis in specific (regular) circumstances and those who consume a lot of eggs as a result of the use of eggs in a lot of dishes and recipes. Agreement with the consumption of eggs in a non-habitual manner would suggest benefit from recipes for novel dishes, coupled particularly with suggestions to increase taste, flavour and variety, to encourage egg intakes in older adults. To include those who prefer a more habitual consumption, simple everyday recipes that involve well known combinations of eggs and other foods, such as bacon or ham in the UK, may be particularly successful. Findings related to lower agreement that eggs may increase cholesterol or risk of heart disease suggest that recent scientific evidence and guidelines against restricting egg consumption [79,80,81] are well known (and/or may be better known in those who consume a lot of eggs), or that those who consume large amounts of eggs failed to acknowledge any previous association, possibly as a result of consuming high quantities of eggs and suffering no health complaints themselves. Various work suggests that older individuals can be less easily swayed by ‘official information’, and can rely more on their own opinions and experiences [82]. These findings suggest that campaigns that continue to address misconceptions that eggs are associated with specific health risks may be beneficial. Findings in relation to a lower willingness to increase egg consumption and greater difficulties stopping eating eggs once started are most likely reversed associations where a high egg consumption results in, as opposed to results from, a lower willingness to increase intakes and a suggestion that it is difficult to stop eating eggs once started.

We also found associations between a higher egg consumption and a higher intake of other protein-containing foods, a younger age, and a higher BMI. These findings suggest that people tend to have either a high intake or low intake of a number of protein-containing foods, including eggs [38,83,84], and strategies to increase protein intake will undoubtably benefit from encouraging intake of a number of protein-rich foods. The consideration of foods or food components that have been linked to increased health risk, such as processed meats, however, would suggest that strategies that focus on *replacing* some of these other protein-containing foods, e.g., with eggs, may also be helpful. Increased consumption of protein-rich foods by those of a younger age, within the older population, has previously been reported [4,30,32,35], and attributed to increased abilities or increased nutritional knowledge in younger individuals [30,32,35]. Associations between egg intake and BMI and a high energy intake have also previously been reported [83,84], although the direction or underlying cause of any association remains unclear [83,84,85,86]. While explanations cannot be disentangled here, strategies aiming to promote eggs to older individuals and to leaner individuals may have an increased chance of benefit on a population-wide basis.

Strengths of our study include the use of a wide population from across the UK and a wide variety of consumers, and the reporting of variable amounts of egg consumption across the sample. The sample was representative of the UK older population based on gender. The under-representation of 55–59 and 80+ years old is likely due to a lack of free time, frailty, and physical or visual impairment. Under-representation of age at both ends of our age range, however, suggests that this is unlikely to have affected our results. The over-representation of individuals in the South West where Bournemouth University is based, and under-representation of Scotland, is also unlikely to have greatly affected our results as egg consumption and attitudes do not differ markedly between regions (personal communication with British Egg Industry Council). Our study is limited by our use of an FFQ to measure intakes of both eggs and protein-containing foods. Frequency measures are commonly used, and have been shown to be valid measures of intake in the elderly [45,46,87,88], but portion sizes were not given nor was it possible to indicate or calculate the number of eggs or amount of protein provided per food item. This may have resulted in differences between respondents in interpretation, but these differences are unlikely to have been systematic. Our final analyses are also limited by the low reliability of some of the components generated by the Principal Component Analysis (Cronbach’s alphas were below 0.5 for five of the 23 generated components). This suggests that the components ‘Social environment’, ‘Time’, and most notably, the components ‘Non-habitual intake’, ‘Willingness to eat more’ and ‘Expectations’ cannot be considered reliable in the current sample, and should be treated with caution.

## 5. Conclusions

In conclusion, many possibilities for future intervention based on existing challenges to or facilitators of egg consumption were found. Our results suggest that strategies to increase egg consumption should focus on: improving liking, tastiness and adding variety; promoting eggs as an everyday type of food; reducing stereotypes about who does and who does not consume eggs; and promoting eggs for people who have noticed the effects of ageing on their food intake. Strategies that highlight value-for-money may also be counterproductive. Future work evaluating the value of these strategies for increasing protein intake in this age group would clearly be of value.

## Figures and Tables

**Table 1:** Participant characteristics (*n* = 230).

Characteristic		Value
Age	55–59 years old	32 (13.8%)
	60–64 years old	38 (16.4%)
	65–69 years old	54 (23.3%)
	70–74 years old	48 (20.7%)
	75–79 years old	36 (15.5%)
	80+ years old	22 (9.5%)
Gender *	Male	119 (51.3%)
	Female	110 (47.4%)
Region	Scotland	11 (4.7%)
	Northern Ireland	5 (2.2%)
	North East	6 (2.6%)
	North West	22 (9.5%)
	Yorkshire and the Humber	21 (9.1%)
	East Midlands	18 (7.8%)
	West Midlands	14 (6.0%)
	Wales	9 (3.9%)
	East of England	17 (7.3%)
	London	17 (7.3%)
	South East	35 (15.1%)
	South West	53 (22.8%)
Marital status *	Married	149 (64.2%)
	Divorced	28 (12.1%)
	Widowed	33 (14.2%)
	Never married	17 (7.3%)
Living status *	Alone	66 (28.4%)
	With others	161 (69.4%)
Education in years (Mean ± standard deviation (SD))		13 ± 2
Most recent employment level *	Unemployed	11 (4.7%)
	Manual worker	44 (19.0%)
	Non-manual worker	86 (37.1%)
	Professional/Management	86 (37.1%)
Body Mass Index (BMI) in kg/m^2^ (Mean ± SD)		27 ± 5
Denture wearing *	No	156 (67.2%)
	Partial dentures	55 (23.7%)
	Full dentures	17 (7.3%)
Receiving help with food shopping *	Never	200 (86.2%)
	Sometimes	19 (8.2%)
	Often	9 (3.9%)
Receiving help with food preparing *	Never	203 (87.5%)
	Sometimes	17 (7.3%)
	Often	8 (3.4%)
Eating out or away from home *	Never	12 (5.2%)
	Sometimes	145 (62.5%)
	Often	68 (29.3%)
Getting food delivered *	Never	162 (69.8%)
	Sometimes	55 (23.7%)
	Often	11 (4.7%)
Physical disabilities *	No	169 (72.8%)
	Some difficulties with some activities	33 (14.2%)
	Some difficulties with all activities	10 (4.3%)
	A lot of difficulty with some activities	7 (3.0%)
	A lot of difficulty with all activities	4 (1.7%)
	Unable to do activities	1 (0.4%)
Food Neophobia score (Mean ± SD)		25 ± 7

* Frequency and percentage are given. For several variables the numbers do not add up to *n* = 230 because different people left different questions open.

**Table 2:** Principal Component Analysis: Component definition and contributing reasons.

Component	Included Reasons	Definition
Liking/flavour/variety (*α* = 0.795)	Variety; Balanced diet; Flavour; Liking	Whether people like the taste of eggs, and think they add variety to the diet (in terms of taste).
Value for money (*α* = 0.715)	Spoilage and wastage (R); Versatility; Standby; Cost; Value; Planning; Complete; Substantial meal; Value for money; Financial situation (R); Nutritional knowledge	Whether people think eggs provide good value for the money you pay for them, including eggs being a good value food, which is cheap and does not go off quickly (not wanting to waste money).
Food chain (*α* = 0.767)	Processing; Freshness; Animal welfare; Wide variety of choice; Quality; Food origin	The importance of knowing about the food chain for the egg, from chicken (animal welfare) to shop to plate, and the quality/freshness of the egg as a result of this.
Everyday food (*α* = 0.686)	Convenience; Satiating effect; Habit (R); Staple food; Recommendations -friends/family; Digestibility	Whether people think eggs are a convenient filling staple food and eat them habitually (including how much this is affected by recommendations from family and friends).
Effort (*α* = 0.700)	Practicalities; Effort to prepare; Politeness; Health beliefs; Culinary skills; Eating abilities; Availability served by others	Whether people think eggs take a lot of effort to prepare, or eat, and would be eaten out of politeness or only when they are served by others. Also, including how healthy people think eggs are.
Previous experience (α = 0.524)	Genes; Previous experience; Medical factors general	Whether people have had a bad experience in the past, or have a family history of problems related to eating eggs, or a medical condition that restricts them from eating eggs.
Past (*α* = 0.595)	Trend availability; Trend popular (R); Upbringing	Whether people had many eggs and remember many people eating them in the past, and/or remember being brought up with eggs.
Occasion (*α* = 0.521)	Comfort; Experience	Whether people eat eggs when there is a particular occasion.
Stereotypes (*α* = 0.565)	Masculinity; Environmental issues; Status personal; Femininity	Amount of agreement with stereotypes or perceptions about a certain type of person who eats eggs.
Sensory (*α* = 0.611)	Odour; Appearance; Texture	Whether people eat eggs for their sensory aspects.
Expectations (*α* = 0.544)	Combination; Status guests; Appeal	How eggs are likely to be eaten in certain circumstances, whether they are suitable with other foods or in certain circumstances, e.g., at a dinner party.
Willingness to eat more eggs (*α* = 0.485)	Sufficiency; Replacing foods	Having clear ideas about the amounts/portions of eggs that are enough, willingness to add more eggs to the diet.
External reports (*α* = 0.483)	Recommendations media; Food scares (R); Recommendations health professionals (R)	How seriously people take external reports and recommendations about eggs and health.
Eating less with aging (*α* = 0.516)	Appetite (R); Sensory abilities; Restraint; Physical abilities shopping; Physical abilities preparing (R)	Whether people suffer from different struggles/problems that may occur when getting older, like physical abilities that hinder shopping or preparing foods, or loss of appetite or sensory deterioration.
Medical factors (*α* = 0.564)	Medical factors cholesterol; Medical factors heart disease	Whether people believe eating eggs increases cholesterol or risk of heart disease.
Time (*α* = 0.448)	Time to prepare; Availability	Whether people perceive eggs as quick to prepare and usually available.
Social environment (*α* = 0.207)	Culture; Other people present; Moral values (R)	Whether people believe eating eggs are part of their society or culture.
Non-habitual intake (*α* = 0.219)	Trying new things (R); Treat	Whether people are willing to consider trying new recipes or would consider eggs as treats.
Moreish (PCA loading = 0.703)	Moreish	Whether people perceive eggs as moreish.
Suitability (PCA loading = 0.744)	Suitability	How suitable it is to eat eggs in a certain context, situation, time, dish, etc. Ideas on how you are supposed to eat them.
Familiarity (PCA loading = 0.796)	Familiarity	The importance of egg dishes people have never tried.
Size (PCA loading = 0.764)	Size	Whether the size of an egg matters.
Food Safety(PCA loading = 0.749)	Food safety	How perceived food safety affects egg intake. Agreement to only eating eggs when they are properly cooked.

* All items ending with (R) were reverse scored, because they had a negative component loading in the Principal Component Analysis (PCA).

**Table 3:** Minimum, maximum, mean and SD for each component for all participants (*n* = 230). Component scores were converted to scores ranging 1–5, to allow comparability with the initial questionnaire response format and coding.

	Minimum	Maximum	Mean	SD
Liking/Flavour/Variety	1.00	5.00	4.03	0.55
Value for money	2.55	5.00	3.89	0.37
Food chain	2.17	5.00	3.70	0.60
Everyday food	1.00	5.00	3.54	0.58
Effort	1.00	4.00	2.13	0.45
Previous experience	1.00	3.67	1.68	0.57
Past	1.00	4.33	2.20	0.80
Occasion	1.00	5.00	2.28	0.81
Stereotypes	1.00	4.00	1.93	0.55
Sensory	1.67	5.00	3.32	0.57
Expectations	1.00	5.00	2.17	0.70
Willingness to eat more eggs	1.00	4.50	2.72	0.72
External reports	1.00	4.67	2.37	0.68
Eating less with aging	1.00	4.00	2.23	0.55
Medical factors	1.00	4.50	2.68	0.80
Time	1.50	5.00	4.08	0.66
Social environment	1.00	4.33	2.81	0.57
Non-habitual intake	1.00	5.00	2.85	0.78
Moreish	1.00	5.00	1.98	0.89
Suitability	1.00	5.00	3.31	0.92
Familiarity	1.00	5.00	3.69	0.74
Size	1.00	5.00	3.22	1.10
Food safety	1.00	5.00	3.76	0.91

**Table 4:** Outcomes of the multiple linear regression model assessing the effect of demographic characteristics and lifestyle factors on egg intake. Significant effects are given in bold (*p* < 0.05).

	*β*	*p*-Value
**Protein intake frequency per month**	**0.307**	**<0.01**
Physical ability score	0.128	0.17
Food neophobia score	−0.093	0.18
Receiving help with food shopping	−0.056	0.56
Receiving help with food preparing	−0.129	0.12
Eating out or away from home	0.019	0.78
Getting food delivered	0.024	0.73
**Age group**	**−0.155**	**0.04**
Gender	−0.079	0.25
**Body Mass Index (BMI)**	**0.157**	**0.02**
Region code	−0.042	0.54
Living status	−0.120	0.09
Years of education	−0.128	0.08
Employment level	0.029	0.69
Denture wearing	0.119	0.10

**Table 5:** Outcomes of the multiple linear regression model assessing the effect of the PCA components, and protein intake, age group and BMI, on egg intake. BMI was imputed with the mean, where necessary. Significant effects are given in bold (*p* < 0.05).

	*β*	*p*-Value
**Liking/Flavour/Variety**	**0.216**	**0.02**
**Value for money**	**−0.254**	**<0.01**
Food chain	0.099	0.16
**Everyday food**	**0.226**	**0.01**
Effort	−0.147	0.08
Previous experience	0.125	0.09
Past	0.036	0.60
Occasion	0.078	0.27
**Stereotypes**	**−0.197**	**0.01**
Sensory	0.030	0.72
**Expectations**	**0.170**	**0.02**
**Willingness to eat more eggs**	**−0.161**	**0.01**
External reports	−0.126	0.08
**Eating less with aging**	**0.250**	**<0.01**
**Medical factors**	**−0.150**	**0.03**
Time	0.017	0.83
Social environment	0.011	0.87
**Non-habitual intake**	**0.142**	**0.04**
**Moreish**	**0.160**	**0.02**
Suitability	0.013	0.83
Familiarity	−0.057	0.37
Size	−0.042	0.51
Food safety	−0.060	0.36
**Protein intake frequency**	**0.238**	**<0.01**
**Age group**	**−0.155**	**0.02**
**BMI**	**0.142**	**0.03**
